# Lessons learnt from the painful shoulder; a case series of malignant shoulder girdle tumours misdiagnosed as frozen shoulder

**DOI:** 10.1186/1477-7800-2-2

**Published:** 2005-01-12

**Authors:** Gerald MY Quan, Derek Carr, Steven Schlicht, Gerard Powell, Peter FM Choong

**Affiliations:** 1 Department of Orthopaedics, St. Vincent's Hospital Melbourne, Australia; 2 Department of Medical Imaging, St. Vincent's Hospital Melbourne, Australia; 3 Division of Surgical Oncology, Peter MacCallum Cancer Institute, Australia

**Keywords:** Frozen shoulder, adhesive capsulitis, hydrodilatation, distension, tumour

## Abstract

Adhesive capsulitis or frozen shoulder is a common condition characterized by shoulder pain and stiffness. In patients in whom conservative measures have failed, more invasive interventions such as arthrographic or arthroscopic distension can be very effective in relieving symptoms and improving range of movement. However, absolute contraindications to these procedures include the presence of neoplasia around the shoulder girdle. We present five cases referred to our institution where the diagnosis of shoulder joint malignancy was delayed, following prolonged, ineffective treatment for frozen shoulder. These cases highlight the importance of careful review of the radiology and the need for reconsideration of the diagnosis in refractory "frozen shoulder".

## Introduction

Frozen shoulder was first described by Codman in 1934, as an idiopathic painful restriction in the range of shoulder joint movement, in the presence of normal plain radiographs [1]. It is also known as "adhesive capsulitis", based on the presence of chronic synovitis and a contracted, thickened joint capsule seen during open surgery of the shoulder joint [2]. It is usually a self-limiting condition, with a mean duration of one to three years [3]. The natural clinical course involves an initial painful phase, followed by progressive stiffness, with a gradual return of functional range of motion [4]. However, between 15 – 50% of patients have persisting severe refractory pain that is unresponsive to conservative management involving physiotherapy, non-steroidal anti-inflammatories and subacromial corticosteroid injections [5,6]. More aggressive treatment options for these patients include manipulation under anaesthesia, arthrographic capsular distension (hydrodilatation), and arthroscopic or open capsular release [7,8]. Hydrodilatation is commonly performed as treatment for frozen shoulder as it is minimally invasive, inexpensive, does not require an anaesthetic, and is effective [9-12]. The procedure involves insertion of a needle into the glenohumeral joint under radiologic guidance, followed by gradual distension of the capsule with a combination of local anaesthetic, corticosteroid and normal saline, until lysis of adhesions and capsular rupture are achieved [13]. Arthrography performed at the beginning of the procedure by injecting radio-opaque contrast material into the shoulder joint is the definitive diagnostic investigation for frozen shoulder, and is associated with decreased joint volume and obliteration of the axillary fold and subscapular bursa.

Tumours around the shoulder girdle are uncommon causes of shoulder pain and stiffness, but often present with symptoms and a clinical history identical to that of a frozen shoulder. A strict contraindication to arthrographic or arthroscopic distension of the shoulder is the presence of a local oncological process. Such procedures may change the surgical management from being a limb-preserving resection to a forequarter amputation. In the past month, five patients have been referred to us with malignant tumours around the shoulder joint, all previously diagnosed as having a frozen shoulder. All patients had undergone prolonged conservative management and hydrodilatation, with persistence of symptoms. Two of the patients had also undergone arthroscopic surgery. The following cases illustrate the importance of reconsidering the diagnosis in refractory frozen shoulder and the value of a detailed clinical history and examination and careful consideration of radiologic imaging in assessing recalcitrant "frozen shoulder".

## Case Reports

### Case 1

**Figure 1:**
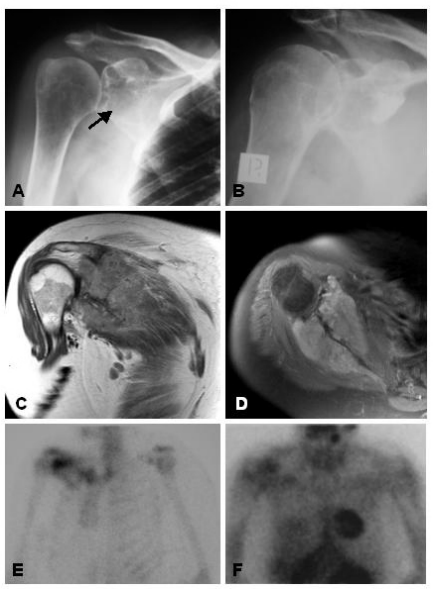
**A **Plain radiograph of the right shoulder, showing an irregular, mixed lytic and sclerotic lesion in the glenoid (arrow), that was not appreciated by the reporting radiologist. **B **Arthrogram performed prior to hydrodilatation. **C **Coronal TSE post-contrast MR image, showing a diffusely enhansive scapular lesion extending into the inferior aspect of the gleno-humeral joint. **D **Axial TSE post-contrast MR image showing diffuse enhancement of the tumour extending on either side of the scapular blade with bony destruction. **E **Bone scan showing increased uptake in the area of the lesion on the delayed static image. **F **Thallium functional scanning showing retained thallium activity in the glenoid region at 4 hrs. Subsequent biopsy was consistent with Ewing's sarcoma.

A 60 year old woman presented to her local medical officer with an eighteen month history of worsening right shoulder pain and stiffness. She was initially treated with oral analgesia followed by a cortisone injection without improvement. Two months later she had a hydrodilatation of the shoulder but her symptoms persisted. MRI was then performed, which demonstrated a large permeative tumour arising from the scapula (Figure 1). She was subsequently referred to us, and underwent staging studies and needle biopsy. Histologic sections were consistent with Ewing's sarcoma.

### Case 2

**Figure 2:**
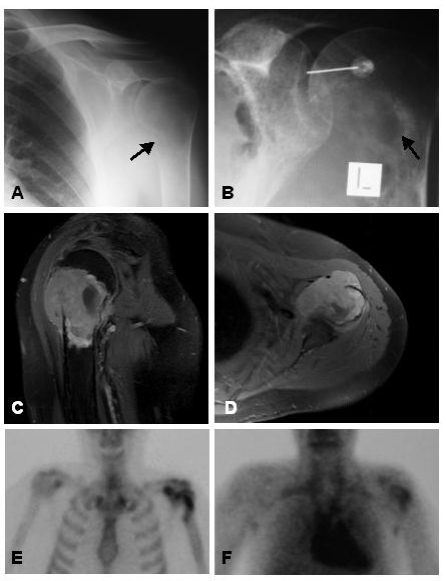
**A **Plain radiograph of the left shoulder showing a lytic lesion affecting the proximal humerus, with cortical irregularity medially (arrow), that was not initially recognized. **B **At the time of arthrographic distension, the lesion (arrow) was more apparent, but remained unnoticed. **C **Sagittal TSE post-contrast MR image showing an enhancing lesion within the proximal humerus extending outside the bone. **D **Axial TSE post-contrast MR image showing the tumour destroying the humeral head and extending into the gleno-humeral articulation. **E **Bone scan showing increased uptake in the area of the lesion on the delayed static image. **F **Thallium functional scanning showing retained thallium activity in the proximal humerus at 4 hrs. Histological sections from the biopsy and surgical resection specimen were consistent with a malignant fibrous histiocytoma.

A 42 year old man was referred to an orthopaedic specialist with a history of sudden onset left shoulder pain following a work related activity. He was initially diagnosed with rotator cuff tendinopathy and subacromial impingement, and had a course of intensive physiotherapy followed by arthroscopic shoulder surgery, without improvement in symptoms. Two months later a minor incident involving his left shoulder led to an increase in pain and swelling and reduction in movement. Hydrodilatation was then performed but pain and function of the shoulder continued to worsen. On retrospective review of plain x-rays of the shoulder, a destructive lesion at the metaphysis with a cortical breach medially in the region of the surgical neck of the humerus was realized (Figure 2). Further anatomic imaging showed an aggressive tumour mass in the proximal diaphysis of the humerus and humeral head extending into the adjacent soft tissues. He was referred to us, and subsequent biopsy of the lesion was consistent with a high-grade pleomorphic sarcoma. After a course of chemotherapy, the patient underwent en bloc resection of the tumour, via an extra-articular approach. Definitive histopathologic diagnosis was malignant fibrous hystiocytoma.

### Case 3

**Figure 3:**
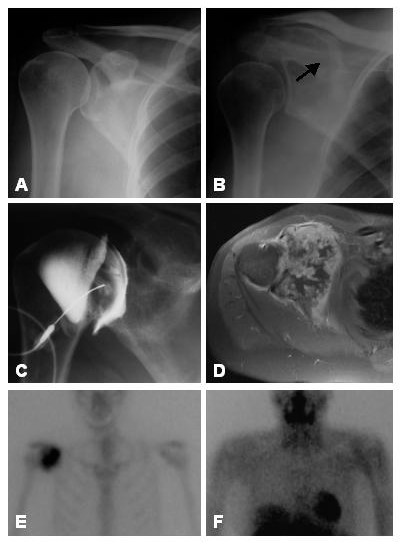
**A **Initial plain radiographs of the shoulder were unremarkable. **B **Repeat radiographs after two years of failed treatment, showing an irregular mixed lytic and sclerotic lesion destroying the coracoid process of the scapula (arrow), which was not appreciated. **C **Arthrogram performed prior to hydrodilatation similarly showing the destructive process, which remained unnoticed. **D **Axial T1-weighted post-contrast MR image showing a heterogenous contrast-enhancing lesion destroying the glenoid and extending into the gleno-humeral joint. The lesion is lobulated and loculated with central areas of lower signal intensity, suggestive of a chondroid lesion. **E **Bone scan showing increased uptake in the area of the lesion on the delayed static image. **F **Thallium functional scanning showing no retention of thallium by the lesion at 4 hrs. Biopsy confirmed low-grade chondrosarcoma.

A 50 year old women was referred to an orthopaedic specialist with a 6 month history of episodic pain in the right shoulder, with associated decreased range of movement. Initial plain x-rays were unremarkable. She then underwent a variety of procedures, which included repeated subacromial corticosteroid injections, arthrographic distension, manipulation under anaesthetic, and arthroscopic debridement and acromioplasty. On arthroscopy, a marked synovitis was observed, to which her ongoing symptoms and the development of a palpable mass on the anterior aspect of the shoulder was initially attributed. Repeat plain radiographs two years after the onset of her symptoms demonstrated a large lesion extending from the glenoid cartilage into the base of the coracoid process (Figure 3). She was then referred to us, where staging radiologic imaging studies and CT-guided biopsy was consistent with a low-grade chondrosarcoma. The patient subsequently underwent en bloc resection of the tumour.

### Case 4

**Figure 4:**
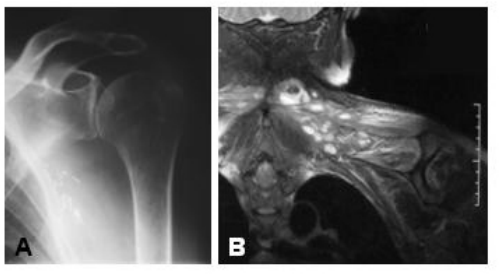
Patient had persistent pain and stiffness following hydrodilatation. **A **Plain shoulder radiographs were normal. **B **STIR MR image showing multiple high signal intensity lesions in the supraspinatus muscle. A presumptive diagnosis of metastatic squamous cell carcinoma was made.

A 68 year old man had previously had a squamous cell carcinoma of the upper back excised, after which he had a three year history of shoulder pain and stiffness. He was treated for a frozen shoulder and received intensive physiotherapy and multiple subacromial corticosteroid injections, followed by hydrodilatation. Initially this seemed to settle his symptoms, although a month later pain and stiffness recurred, with marked reduction in shoulder function, and he was referred to us. An MRI was performed, which showed lesions in the supraspinatus and trapezius muscles, which were consistent with metastatic deposits (Figure 4). The patient underwent a course of palliative radiation therapy.

### Case 5

**Figure 5:**
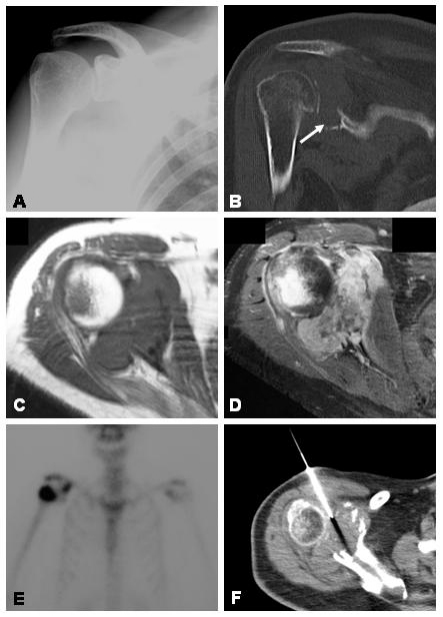
**A **Initial plain radiographs of the right shoulder appeared normal. **B **CT scan was performed after a year of progressive shoulder pain and stiffness, showing a destructive lesion involving the glenoid (arrow). **C **Axial T1-weighted and post-contrast (**D**) MR images showing destruction of the glenoid with an associated soft tissue mass and involvement of the humeral head. **E **Bone scan showing increased osteoblastic activity involving the coracoid process and right humeral head and relative photopaenia of the glenoid. **F **CT-guided percutaneous biopsy was able to obtain a histological diagnosis of malignant fibrous histiocytoma.

A 55 year old female with a past history of malignant fibrous histiocytoma of the left thigh resected five years previously, presented to her local medical officer with right shoulder pain. Plain films were performed at the time which appeared normal (Figure 5). Subsequent treatment included multiple intra-articular injections of corticosteroid and local anaesthetic, however her pain and associated restricted movement worsened. She was referred for an orthopaedic surgical opinion and shoulder ultrasound. An arthroscope was performed which demonstrated rotator cuff pathology but failed to reveal the actual cause of the patient's symptoms. One year after the onset of her original symptoms, repeat plain films showed destruction of the glenoid and coracoid process. A bone scan demonstrated increased osteoblastic activity involving the coracoid process and right humeral head and relative photopaenia of the glenoid. Functional thallium scintigraphy showed increased metabolic activity around the right shoulder joint with CT and MRI scanning confirming destruction of the glenoid with an associated soft tissue mass and involvement of the humeral head (Figure 5). CT-guided percutaneous biopsy was performed and diagnosis of malignant fibrous histiocytoma made.

## Discussion

Adhesive capsulitis or frozen shoulder is a common condition that may affect up to 5% of the general population in their lifetime. Although the aetiology of frozen shoulder is unknown, it has been associated with diabetes mellitus, thyroid disease, ischaemic heart disease and various autoimmune conditions [14]. Other causes of shoulder pain and stiffness that need to be excluded include rotator cuff pathology, arthritis, fractures, infection and local tumours [15,16]. Arthrographic or arthroscopic distension with shoulder capsular rupture are effective treatment modalities in well-selected patients with refractory frozen shoulder symptoms despite intensive conservative management [5-7]. In a recent randomised, double blinded study, Buchbinder *et al*. [12] demonstrated a significant improvement in both pain and range of motion in patients treated with hydrodilation compared with arthrogram alone. However, absolute contraindications to surgical intervention for frozen shoulder include neurological abnormalities originating from the cervical spine, presence of infection, and an ongoing oncological process.

Tumours of the shoulder girdle are uncommon causes of shoulder pain and restricted movement. In most cases, they are diagnosed based on the presence of a soft tissue mass on clinical examination, as well as characteristic radiographic changes. Robinson *et al*. [17] suggested that younger patients with bony tenderness elicited by gentle tapping are more likely to have a shoulder neoplasm. However, in up to 10% of shoulder neoplasms, plain x-rays are normal, and these patients may present with painful limitation of shoulder motion that can be difficult to distinguish from primary frozen shoulder. Indeed, in one series of 140 patients with frozen shoulder referred for manipulation, 2% had a primary chest wall tumour [18]. Misdiagnosis, inappropriate surgery and delayed therapy for shoulder symptoms due to malignancy may potentially have grave consequences. Our five patients had locally invasive malignant tumours, and received prolonged conservative and interventional treatment for "frozen shoulder" before the definitive diagnosis of tumour was made. In all cases of recalcitrant frozen shoulder resistant to conventional treatment, less common causes for shoulder pain and stiffness such as an ongoing oncological process must be considered. A detailed clinical history and examination is critical in the assessment of a painful, stiff shoulder. Plain antero-posterior and axillary lateral radiographs of the shoulder should be performed as a routine, and these films then require careful review by an experienced radiologist prior to undertaking any invasive procedures. More sensitive radiological investigations such as radionucleotide scanning and CT scanning or MRI should be considered when shoulder symptoms are atypical or progress despite invasive management, if there is suspicion of malignancy, or if there are any bony abnormalities evident on plain radiographs.

## Abbreviations

MRI: magnetic resonance imaging, CT: computed tomography, TSE: turbo spin echo, STIR: short tau inversion recovery.
